# Slowly repeated evoked pain (SREP) as a central sensitization marker in episodic migraine patients

**DOI:** 10.1038/s41598-021-84157-1

**Published:** 2021-02-25

**Authors:** Pablo de la Coba, Stephen Bruehl, Gustavo A. Reyes del Paso

**Affiliations:** 1 Department of Psychology, University of Jaén, 23071 Jaén, Spain; 2 Department of Anesthesiology, Vanderbilt University Medical Center, Nashville, TN USA

**Keywords:** Psychology, Health care, Rheumatology

## Abstract

Migraine headache is a pain condition characterized by severe and recurrent unilateral head pain. Among other mechanisms, central pain sensitization processes seem to be involved in the disorder. An experimental protocol based on slowly repeated evoked pain (SREP) has been shown to indicate pain sensitization in fibromyalgia patients and differentiate these patients from healthy individuals and rheumatoid arthritis patients. This study examined SREP sensitization in migraine patients and explored its potential usefulness as a central sensitization marker. The SREP protocol was administered to 40 episodic migraine (EM) patients not currently experiencing a headache and 40 healthy controls. SREP consisted of a series of 9 suprathreshold painful pressure stimuli of 5 s duration and a 30 s interstimulus interval. SREP sensitization was indexed by the increase in pain ratings across the stimuli. Pain threshold, pain tolerance and temporal summation of pain were also assessed. SREP sensitization was observed in EM, but not in healthy individuals (*p* < .001). SREP differentiated between EM and healthy individuals with up to 75% diagnostic accuracy. Pain threshold, pain tolerance and temporal summation of pain did not show significant discriminative ability. An SREP index value of 0.5 was the most sensitive cut-off for detecting central pain sensitization when prioritizing diagnostic sensitivity (0.88). Results provide evidence for SREP as a possible central sensitization marker with potential clinical utility in migraine patients. Inclusion of SREP in Quantitative Sensory Testing protocols may enhance the assessment of altered pain modulation in different pain conditions.

## Introduction

Migraine is a neuro-vascular disease characterized by severe, throbbing and recurrent unilateral pain. According to the World Health Organization, it is the third most prevalent disease worldwide^1^. Despite this, its underlying mechanisms are not yet fully understood^2^. Both central and peripheral sensitization related to the activation of trigeminovascular neurons seem to be involved in the different pain components of migraine^3^. Although it remains unclear whether central nervous system (CNS) alterations are a cause or consequence of migraine^2^, some authors have suggested that central sensitization mechanisms similar to those noted in fibromyalgia may also be involved in migraine^4,5^.

Central sensitization (CS) can be broadly defined as an amplification of pain responses, reflected in hypersensitivity, allodynia or hyperalgesia in response to peripheral inputs, that is produced by a CNS-related upregulation of ascending facilitatory pain pathways (bottom-up regulation) and/or an impairment of descending inhibitory pain mechanisms (top-down regulation)^6^. Experimental evidence of CS in migraine has been observed both during and between attacks^7–9^. Nonetheless, additional mechanisms like peripheral sympathetic nervous system, hypothalamo-pituitary-adrenal axis or immune system changes also seem to be involved in the pathophysiology of CS-related syndromes like migraine^10,11^.

The measurement of CS has been addressed in different ways, ranging from invasive methods used in animal pain models^12,13^, to non-invasive methodologies used in human research^14–16^. These non-invasive CS measures include neuroimaging of responses to painful stimuli^16–19^, evoked pain measures^20,21^, and self-administered questionnaires assessing CS-related symptoms^22,23^. One of the techniques most commonly used to examine CS is Quantitative Sensory Testing (QST), which is based on examining responses to various standardized evoked pain stimulation protocols^24,25^.

The utility of evoked pain indices for the characterization and discrimination of different chronic pain conditions has been demonstrated using a range of different evoked pain protocols^26,27^. Evoked pain protocols can be classified into static and dynamic^28,29^. Static protocols evaluate pain responsiveness under basal state conditions of the nociceptive system while dynamic protocols evaluate pain modulation by examining changes in perceived pain in the context of prior stimulation of the nociceptive system, such as repeated pain stimuli, and can assess function specifically in the ascending facilitatory or descending inhibitory pain pathways depending on the protocol^30,31^. Assessment of capacity to modulate pain is best carried out through use of dynamic evoked pain indices, rather than typical static evoked pain measures like pain threshold and tolerance^32–34^. In this regard, the dynamic evoked pain indices most widely studied have been Conditioned Pain Modulation (CPM), which reflects descending pain inhibition^35^; and Temporal Summation of Pain (TSP), which shows ascending pain facilitation believed to reflect the “wind-up” effect in the spinal cord^36^.

In migraine, CPM has been found to be reduced or absent^37^ and TSP augmented^9^. However, current evidence is not entirely consistent^38^, suggesting the involvement of additional factors in this condition. Nonetheless, it seems clear that alterations in pain modulation are present in many migraine patients, including CS to pain^39^. It has been proposed that migraine and fibromyalgia may share common pathogenic mechanisms at the CNS level^5,40,41^. For example, lower CPM^42^ and higher TSP^43^ have also been found in fibromyalgia, although contradictory findings are also available^44,45^. In addition, both conditions have shown a notably greater prevalence in women than men^46,47^.

In our previous work, a Slowly Repeated Evoked Pain (SREP) protocol, consisting of 9 suprathreshold pressure painful stimuli (adapted to individual pain sensitivity) applied to the finger nail, each 5 s in duration and with a 30 s inter-stimulus interval, was identified as a potential CS marker independent of TSP. Like TSP, SREP is a dynamic protocol that assesses changes in pain perception in response to repeated evoked pain stimuli, but with stimuli presented at a much lower frequency not believed to elicit TSP^48,49^. While fibromyalgia patients displayed SREP sensitization, healthy individuals did not, with SREP showing an 85.4% diagnostic accuracy in discriminating these groups^50^. The SREP index showed similar ability to differentiate between patients with fibromyalgia and rheumatoid arthritis. SREP sensitization was not observed in rheumatoid arthritis patients (a predominantly peripheral pain condition), whereas it was present in fibromyalgia patients (an accepted central pain condition). Therefore, although the underlying mechanisms of SREP remain unknown, these findings suggested an involvement of central rather than peripheral pain sensitization processes in the SREP phenomenon^51^. The SREP protocol has also shown good reliability and appears to be clinically relevant given its association with clinical pain^50,51^. However, it remains unclear whether SREP is a specific fibromyalgia marker or rather a more general index of CS-related nociceptive alterations that may be relevant to other pain conditions believed to have a central component, such as migraine.

Given the accuracy of the SREP index in differentiating between fibromyalgia patients and both healthy individuals and patients with rheumatoid arthritis^50,51^, we hypothesized that SREP might also have potential utility as a general CS marker in other pain conditions hypothesized to have a centralized component. Migraine headache appeared to be a suitable candidate, since TSP has been observed in some studies with migraine patients^20,52^ and evidence suggests possible shared pathogenic mechanisms with fibromyalgia^5,40,41^. In this context, the aims of the present study were threefold: (I) to explore the SREP sensitization response in migraine patients; (II) to examine the capacity of SREP to discriminate migraine patients from healthy individuals; and (III) to compare SREP pain responses to the most frequently used static (pain threshold and tolerance) and dynamic (TSP) evoked pain indices. We hypothesized that SREP sensitization would be observed in migraine patients and not in healthy participants, that the SREP index would accurately discriminate migraine patients from healthy participants, and that this discrimination accuracy would be substantially greater for SREP than for the more commonly used TSP and pain threshold and tolerance measures.

## Methods

### Participants

Forty female university students with migraine without aura in the interictal phase participated in the study. No migraine attacks or other headaches were present for at least 48 h prior to study. Headaches were diagnosed by a neurologist according to the 3rd version of International Headache Society (IHS) criteria (2018)^53^. All participants reported a frequency of 4–14 days with migraine attacks/month, which is commonly known as “episodic migraine” (EM; in contrast to chronic migraine). Forty healthy female university students, matched for age and body mass index, were included as a control group in the study. All participants were aged between 18 and 30 years old. Exclusion criteria were the presence of any other neurologic or cardiovascular disease, metabolic abnormalities, or any psychiatric disorder or comorbid pain conditions, including other headaches with the exception of tension-type headache occurring less than once per month. Presence of medication-overuse headache was excluded by requiring that participants be using analgesic medications less than 10 days/month. Clinical and demographic data for the sample can be found in Table 1. All participants provided written informed consent.

**Table 1:** Demographic, clinical, and evoked pain data by group for episodic migraine patients and healthy individuals.

Variables	EM (n = 40)	Healthy (n = 40)	EM vs Healthy		
			*t* or* χ*^2^	p**	η^2^
Age (years)	20.95 ± 3.05	21.25 ± 3.45	− 0.412	0.682	0.002
BMI	23.04 ± 3.52	22.09 ± 3.36	1.244	0.217	0.019
Pain threshold (kg)	3.38 ± 1.45	3.18 ± 1.76	0.553	0.582	0.004
Pain tolerance (kg)	7.99 ± 1.92	7.99 ± 2.19	0.004	0.997	< 0.001
SREP sensitization (VAS) [0–10]	1.00 ± 0.62	− 15 ± 0.98	5.544	< 0.001***	0.283
SREP stimulus pressure (kg)*	4.82 ± 1.36	4.68 ± 1.76	0.391	0.697	0.002
TSP (NRS) [0–10]	0.43 ± 0.72	0.56 ± 1.12	− 0.654	0.515	0.005
Present Pain Index (MPQ) [0–5]	0.45 ± 0.86	0.35 ± 0.58	0.602	0.549	0.005
First pain rating SREP (VAS) [0–10]	3.25 ± 1.86	3.47 ± 1.94	− 0.518	0.606	0.003
First pain rating TSP (NRS) [0–10]	1.44 ± 1.29	1.31 ± 1.34	0.424	0.673	0.002
Catastrophizing (CSQ) [0–36]	15.28 ± 7.37	7.23 ± 5.58	5.509	< 0.001***	0.280
Anxiety (HADS-A) [0–21]	8.30 ± 2.95	8.18 ± 3.10	0.185	0.854	< 0.001
Depression (HADS-D) [0–21]	3.78 ± 3.00	4.38 ± 3.46	− 0.828	0.410	0.009
Regular analgesics use, N (%)	24 [60%]	0 [0%]	34.286	< 0.001***	0.002
Age of onset of migraine (years)	11.98 ± 3.62	–	–	–	–
Intensity of migraine attacks [0–10]	7.68 ± 1.00	–	–	–	–
Duration of migraine attacks (h)	10.15 ± 6.10	–	–	–	–
Frequency of migraine (days/month)	6.68 ± 3.74	–	–	–	–

Means ± Standard Deviations, and results of the group comparisons (*t* or* χ*^2^, p, and η^2^).

*Individually calibrated stimulus pressure for the SREP procedure in kg/cm2.

**Original *p*-values from group comparisons.

***Significant *p*-values after Bonferroni adjustment remained below 0.001.

*BMI* Body Mass Index, *CSQ *Coping Strategies Questionnaire, *EM *Episodic Migraine, *HADS *Hospital Anxiety and Depression Scale, *MPQ *McGill Pain Questionnaire, *NRS *Numeric Rating Scale, *SREP *Slowly Repeated Evoked Pain, *TSP *Temporal Summation of Pain, *VAS *Visual Analogic Scale.

### Assessment clinical and psychological factors

The following self-report questionnaires were used:

- The Present Pain Intensity index of the McGill Pain Questionnaire (PPI-MPQ)^54^. The PPI-MPQ consists of a simple 6 category assessment of current clinical pain (from “No Pain” to “Excruciating”). Its score range was between 0 and 5^55^. It was used to assess the intensity of any current clinical pain.
- The catastrophizing subscale of the Coping Strategies Questionnaire (CSQ)^56^; Spanish version^54^. The CSQ is a self-administered questionnaire composed of different subscales that measure cognitive and behavioral pain coping strategies. The catastrophizing subscale consisting of 6 items with a score range from 0 to 36 and internal consistency of alpha = 0.89^57^.
- Hospital Anxiety and Depression Scale (HADS)^58^; Spanish version^59^. The HADS was designed to assess anxiety and depression while minimizing the influence of biological symptoms^58^. Each subscale consists of 7 items, with a score range from 0 to 21 and internal consistency of alpha = 0.86^60^.

### SREP protocol

As part of determining stimulus intensity for the SREP protocol, static evoked measures of pain threshold and tolerance were obtained. Pain threshold and tolerance concepts were explained in advance to participants as “the lowest pressure stimulation that causes pain” and “the highest pressure stimulation that you are able to tolerate”, respectively. Once these concepts were understood, both measures were obtained using the algometer at an increasing pressure rate of 1 kg/s on the third fingernail of the left hand. To obtain the pain threshold measure, the increasing pressure was applied until each participant reported verbally that pressure had become painful. Two minutes later, the pain tolerance measure was obtained using the same stimulus protocol, but with each participant reporting verbally when the stimulus achieved the maximal tolerated pressure.

The SREP protocol was applied in the same way as in all of our previous work^50,51,61^. It consisted of a single series of 9 low-to-moderate intensity painful stimuli applied to the third finger nail of the left hand for 5 s duration and with an interstimulus interval of 30 s. A pressure algometer Tracker Freedom (JTECH Medical, Lawndale, USA) with a stimulation surface area of 1 cm^2^ was used to apply the pain stimuli. The intensity of painful stimulation for the SREP protocol was calculated individually based on the measures of pain threshold and tolerance for each participant according to the following formula: Intensity = Threshold + 1.25 × (DF/4); where DF = Tolerance − Threshold^62^. The pain experienced in response to each stimulus was evaluated using a 10 cm visual analog scale (VAS) with the following anchors: “No Pain” and “Extremely Painful.” Five seconds after termination of each pain stimulus, the VAS was presented to the participant. Twenty seconds after each VAS assessment, the subsequent SREP stimulus was delivered. In this way, the duration for the complete administration of the SREP protocol was approximately 4.5 min. Finally, the SREP index was calculated as the change in pain response between the ninth and the first VAS pain rating (larger positive values indicate a greater SREP response).

### TSP protocol

The TSP protocol was applied using the protocol describe by Goodin et al. (2014), through use of a nylon monofilament (Touchtest Sensory Evaluator 6.65) calibrated to bend at 300 g of pressure^63^. Two TSP series of 10 applications of this stimulus at a rate of 1 Hz (1 touch/second) were delivered on the thenar eminence of the left hand. The resting interval between the two series of TSP stimuli was 30 s. The subjective pain intensity of each touch was assessed via verbal response through a 0–10 Numeric Rating Scale, with “0” being “no pain” and “10” being “extremely painful.” A summary TSP index was derived as the difference between the last and the first pain rating (larger positive values indicate greater TSP), with the average of the two TSP series used as the TSP index in analyses. This TSP protocol has been successfully used to assess CS in patients with knee osteoarthritis^63^ and both fibromyalgia and rheumatoid arthritis^51^.

### Procedure

Participants were recruited from the pool of students seeking psychology and teaching degrees of the University of Jaén (Spain). For EM participants, the first step was verifying the diagnosis provided by the treating neurologist through the review of medical records. The study was performed in a single session of approximately 60 min in duration that was divided in three different parts:

(I) *Clinical interview* A psychologist obtained informed consent, checked the inclusion–exclusion criteria, obtained the participant´s clinical history through a semi-structured interview that addressed (a) sociodemographic data, (b) clinical features of migraine attacks such as age of onset (years), intensity (numeric rating from 0 to 10), frequency (days/month), and average duration (hours) and (c) regular analgesic use (acetaminophen, naproxen, aspirin and/or ibuprofen), with the self-report questionnaires administered immediately following the interview.
(II) *SREP protocol* First, pain threshold and tolerance concepts were explained to participants, and then both were obtained according to the protocol described above. Next, over a 5 min period, VAS pain assessment procedures were practiced through a series of 3 stimuli of 5 s duration and different intensities (1.5, 2.5, and 2.0 kg/cm^2^ in sequence) applied to the second finger nail of the left hand. Finally, the SREP protocol described above was administered using the protocol previously described.
(III) *TSP protocol* First, instructions were provided and a brief practice series of stimuli to train participants in the NRS pain rating procedures were carried out on the right hand to ensure ability to provide a verbal pain rating once per second. Following this, the two series of TSP stimuli were administered on the left hand according to the protocol described above.

SREP and TSP protocols were administered in a randomized-counterbalanced order, with a 5 min rest period between protocols. Participant type was also counterbalanced, i.e. the examiner evaluated migraine patients and healthy individuals in an alternating order. The investigator was not blinded to participant type. All participants were asked not to consume alcohol or caffeine, and not to engage in intense physical exercise for 24 h before the study. Before proceeding with laboratory procedures, the investigator confirmed that EM patients did not experience any migraine attacks or use analgesic drugs for at least 48 h prior to the study. The study was approved by the Ethics Committee of the University of Jaén, and it is line with the updated declaration of Helsinki^64^.

### Statistical analyses

In order to determine the optimal sample size based on expected effect sizes, the G*Power 3.1.7 program (University of Düsseldorf) was used^65^. Previous comparisons between fibromyalgia patients and healthy individuals on the SREP index showed large effect sizes, with Cohen´s d in the range 1.42–1.79^34,50,61^. Assuming an effect size of 1.6 and an alpha level of 0.05, a sample size of 40 participants per group was determined to be sufficient to result in a power of 0.80.

All analyses were conducted using the SPSS for Windows Version 19 statistical package (IBM Corp., Armonk, NY). Kolmogorov–Smirnov and Levene’s tests showed no deviations from normality or homogeneity assumptions in any of the targeted variables. Student's t-tests for independent samples were performed to compare demographic, clinical and evoked pain data between groups (Table 1). To provide an optimal analysis of dynamic evoked pain indices given the repeated nature of the stimuli, the patterns of pain responses to the SREP and TSP protocols were examined using repeated-measures ANOVA with one between-subjects factor (Group: migraine vs. healthy) and one repeated-measures factor (the nine VAS pain ratings for SREP, and the ten NRS pain ratings for TSP). To rule out the possibility that clinical variables could confound these results, we repeated these primary analyses entering the clinical variables that showed group differences as covariates.

The Greenhouse–Geisser procedure was used to correct for degrees of freedom in repeated measures analyses. Results are reported with the original degrees of freedom and the corrected *P* values. Effect sizes were reported as adjusted eta-squared (η^2^). Associations between dynamic indices (SREP and TSP) and catastrophizing and clinical pain, associated with SREP in our previous study with FM patients^50^, were tested using Pearson correlations. Results of correlations and group comparisons in demographic and clinical variables were adjusted for multiple comparisons using Bonferroni procedure.

A series of logistic regression analyses were next conducted to determine the diagnostic accuracy of static evoked pain measures (Threshold + Tolerance), TSP, and SREP sensitization for differentiating between EM and healthy participants. Sensitivity and specificity indices were derived from the resulting actual vs predicted group memberships. Sensitivity indicated the proportion of EM patients correctly detected (true positives vs. false negatives) while specificity indicated the proportion of healthy individuals correctly classified (true negative vs. false positives).

Finally, Receiver Operating Characteristic (ROC) analyses were performed to determine the most accurate cut-off points for differentiating EM and healthy individuals using the SREP index. According to the procedures described by Altman & Bland (1994)^66^, ROC curves were created by plotting the range of each participant’s error rate, with clinical or non-clinical status (EM vs. healthy) as the classifier variable. In order to obtain these sensitivity and specificity pairs, the SPSS ROC curve procedure was used, entering the SREP index as the Test variable and Group as the State variable, with the “Coordinate points of the ROC curve” option selected. Cut-off points were estimated using the best combinations according to Youden's J statistic (J = Sensitivity + Specificity − 1). The cut-offs with (a) higher Youden index, and (b) sensitivity and specificity greater than 60%, were selected. Area under the ROC curve (AUC) was used to obtain another estimate of the diagnostic accuracy of SREP.

### Ethical standards

This study was approved by the Ethics Committee of the University of Jaén according to the declaration of Helsinki of 1964, as revised in 2013.

## Results

### Group differences in demographic, clinical and evoked pain responses

There were no demographic differences between EM and healthy participants. For clinical and evoked pain variables, there were significant group differences in catastrophizing and analgesic use, with greater levels in EM patients than healthy participants. SREP sensitization responses also differed between groups, with SREP being observed in the EM group (SREP index > 0) but not in healthy participants (SREP index ≤ 0). No significant group differences were found for pain threshold, tolerance, or TSP (see Table 1).

### SREP sensitization

**Figure 1:**
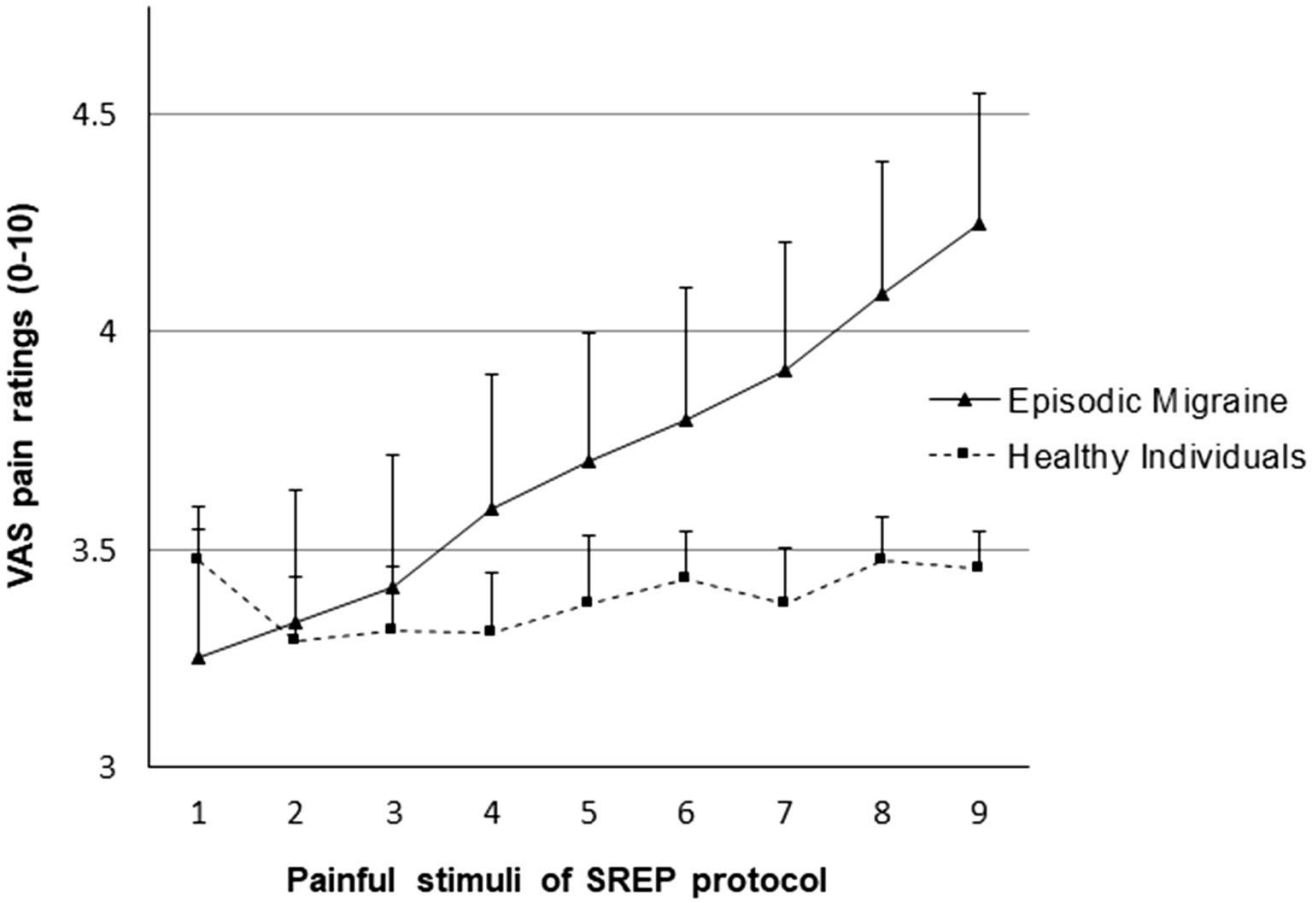
Mean (+ SE) evoked pain ratings (0–10 scores) over repeated stimuli as a function of group across the 9 painful stimuli of the SREP protocol.

Pain intensity ratings increased overall as the trials progressed in the SREP protocol (main effect of trials: F(8,624) = 21.71, p < 0.0001, η^2^ = 0.22). However, this effect differed as a function of group (group × trial interaction: F(8,624) = 14.16, p < 0.0001, η^2^ = 0.15). Simple effects analyses revealed that pain intensity ratings increased progressively in EM (F(8,312) = 48.64, p < 0.0001, η^2^ = 0.56), but not in healthy participants (F(8,312) = 1.21, p = 0.31, η^2^ = 0.03). Figure 1 displays the patterns of pain ratings during the SREP protocol by group. There were no group differences in the overall perceived intensity of evoked pain between EM and healthy groups (between-subjects main effect of group: F(1,78) = 0.55, p = 0.46, η^2^ = 0.01). Group comparisons performed on a "stimulus-by-stimulus" basis for the nine VAS pain ratings of the SREP series indicated that even the ninth pain rating was not significantly different between EM and healthy individuals (p = 0.068). Thus, results for SREP confirm that the dynamics of changes in pain response over time are the critical issue rather than differences in perceived intensity of individual stimuli. Only current clinical pain intensity (PPI-MPQ) correlated positively with the SREP index in EM patients (*r* = 0.39, *p* = 0.013, Bonferroni adjusted p = 0.026).

To rule out potential confounds that might alter interpretation of SREP effects including catastrophizing and regular analgesics use (higher in the EM group), and clinical pain intensity (the PPI-MPQ was associated with SREP), we repeated the primary analyses above including these variables as covariates. These analyses failed to reveal any significant effects of the covariates (all p’s > 0.12), and the previous significant results were maintained.

### Temporal summation of pain

**Figure 2:**
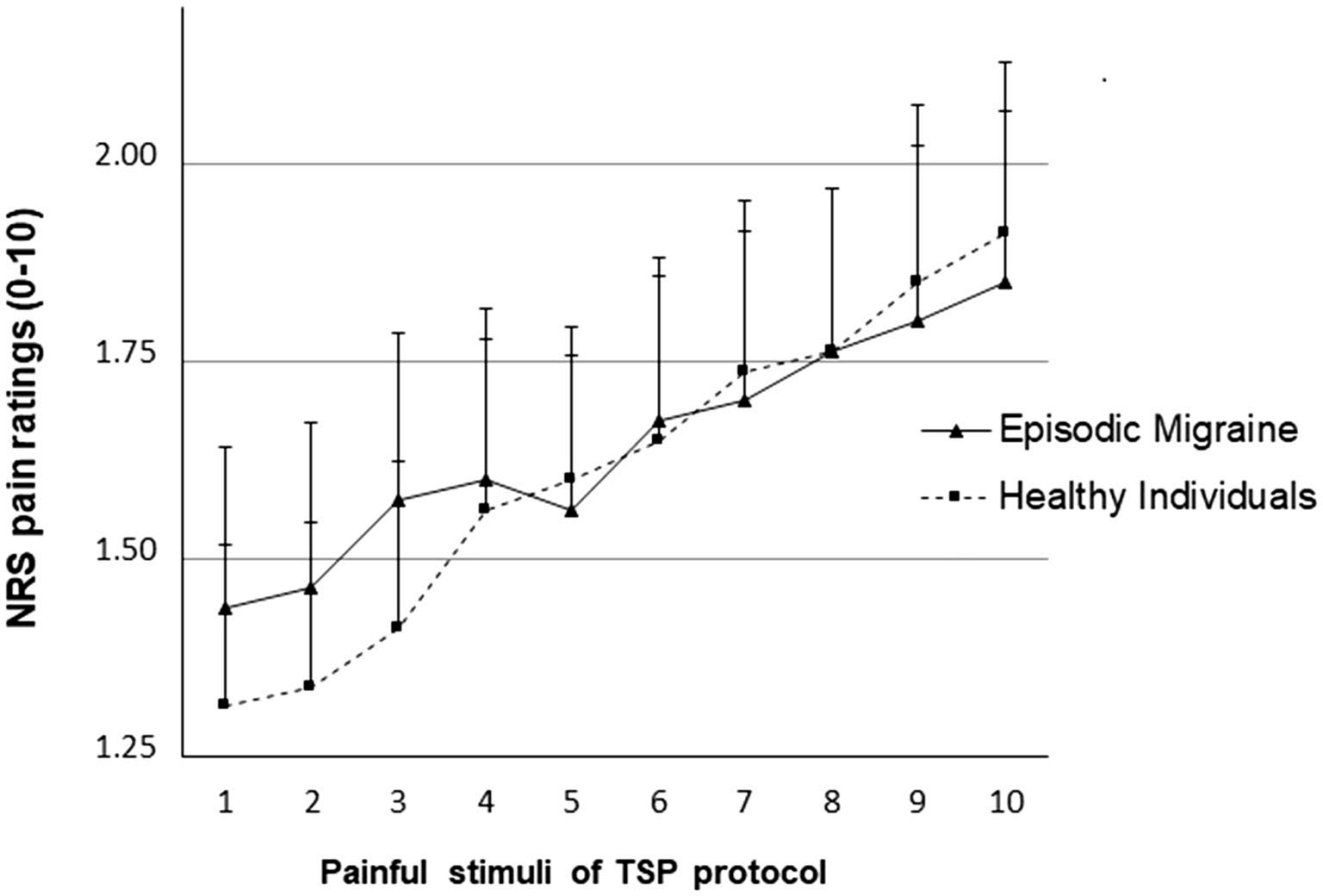
Mean (± SE) NRS pain ratings (0–10) over repeated stimuli as a function of group across the 10 painful stimuli of the TSP protocol.

Pain intensity ratings increased as the trials progressed in the TSP protocol (main effect of trials: F(9,702) = 13.94, p < 0.0001, η^2^ = 0.15) with no differences as a function of group (group × trial interaction: F(9,702) = 0.79, p = 0.48, η^2^ = 0.01). Finally, there was no differences in the overall perceived intensity of evoked pain in the TSP protocol between EM and healthy groups (between-subjects effect of group: F(1,78) < 0.01, p = 0.46, η^2^ = 0.01). Figure 2 displays the pattern of average pain ratings across trials during the TSP protocol in both groups. Neither catastrophizing nor PPI-MPQ were correlated with TSP (all *p’s* > 0.16, Bonferroni adjusted p’s > 0.033).

To rule out catastrophizing and regular analgesics use (higher in the EM group) as potential confounds to TSP effects, we repeated the primary analyses above including these variables as covariates. These analyses failed to reveal any significant effects of catastrophizing or regular analgesic use (p’s > 0.66), and they did not alter the pattern findings reported above.

### Diagnostic accuracy of evoked pain measures

Logistic regression analyses conducted to differentiate between EM patients and healthy participants showed that only SREP discriminated significantly between groups (β =  − 2.48, SE = 0.67, Wald = 13.91, p < 0.001). Neither the static pain measures (pain threshold: β =  − 0.12, SE = 0.18, Wald = 0.47, p = 0.495; pain tolerance: β = 0.54, SE = 0.14, Wald = 0.16, p = 0.692) nor TSP (β = 0.25, SE = 0.28, Wald = 0.75, p = 0.385) discriminated significantly between the EM and control groups. Sensitivity and specificity values derived from these logistic regression analyses are summarized for each evoked pain measure in Table 2. Overall accuracy of discrimination between groups was notably higher for SREP (71.3%) than for the other QST measures (≤ 52%), with an overall sensitivity and specificity ≥ 0.70 observed for the continuous SREP index.

**Table 2:** Sensitivity and specificity values and overall accuracy for the discrimination between EM and healthy individuals using pain threshold + tolerance (combined), Temporal Summation of Pain (TSP), and Slowly Repeated Evoked Pain (SREP) sensitization.

	Threshold + Tolerance	TSP	SREP sensitization
Sensitivity	0.50	0.57	0.70
Specificity	0.53	0.38	0.73
Overall accuracy	51.3%	47.5%	71.3%

### Classification cut off points for SREP

**Figure 3:**
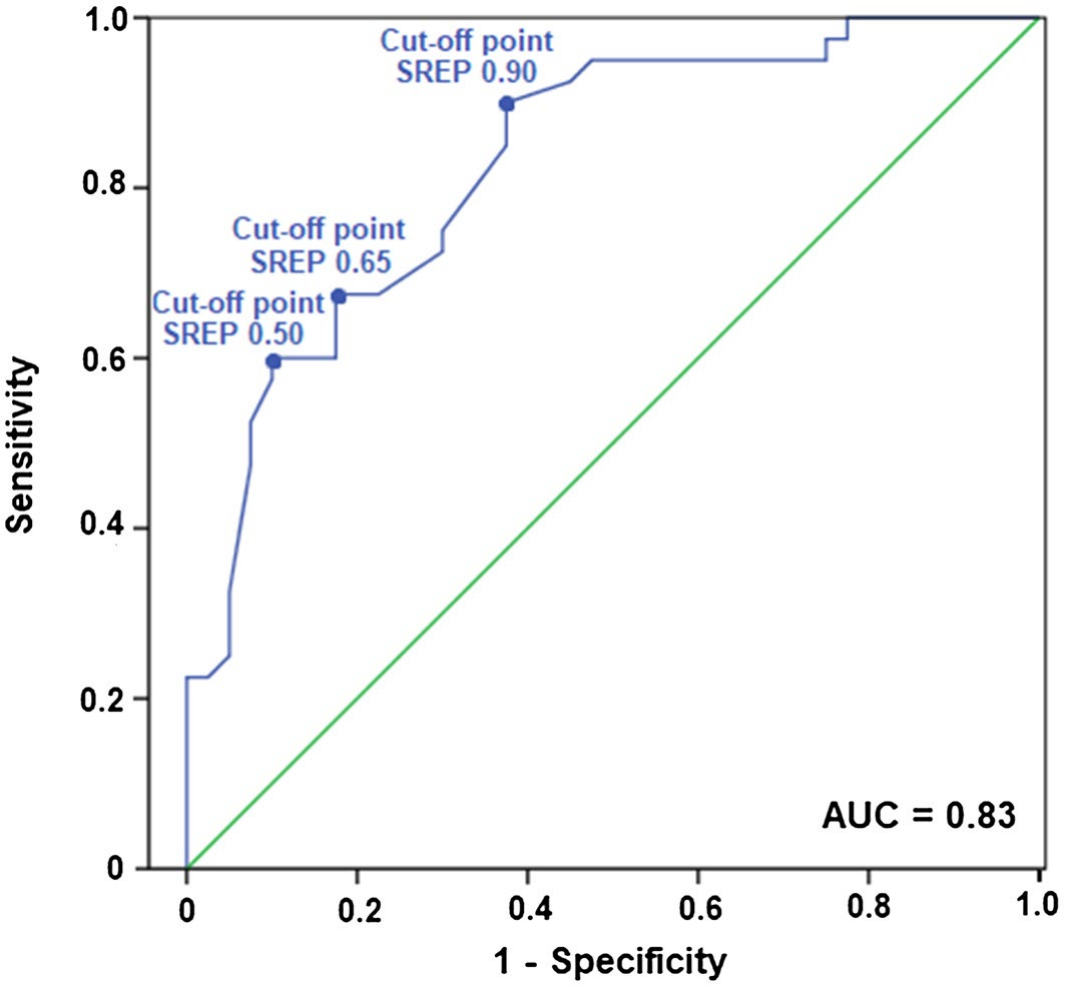
Receiver operating curve for the SREP index.

Based on the maximum achieved value of the Youden index (J = 0.50) derived from ROC analyses of SREP and the minimum criteria for sensitivity and specificity (both required to be > 60%), three SREP values were identified as the most sensitive cut-off points to discriminate correctly between patients with EM and healthy individuals. These three cut-off points, together with their associated sensitivity and specificity values, are presented in Table 3. In addition, based on the AUC interpretation guidelines of Hosmer et al.^67^, the AUC resulting from the ROC analyses (see Fig. 3) further corroborated the excellent discriminatory power of the SREP index (AUC = 0.83, SE = 0.05, p < 0.001, 95% CI 0.74–0.92).

**Table 3:** Sensitivity and specificity values, overall accuracy and Youden index for the three selected SREP cut-off points (0.50, 0.65 and 0.90) for discriminating between EM and healthy individuals.

	SREP 0.50	SREP 0.65	SREP 0.90
Sensitivity	0.88	0.68	0.60
Specificity	0.62	0.82	0.90
Overall accuracy (%)	75%	75%	75%
Youden Index (J)^a^	0.50	0.50	0.50

*SREP *slowly, repeated evoked of pain.

^a^Youden Index (J) = Sensitivity + Specificity − 1.

## Discussion

The present study tested the hypothesis that a dynamic evoked pain index based on application of slowly repeated evoked pain stimuli (SREP) might serve as a broad index of CS sensitization across a variety of pain conditions hypothesized to be CS-related, such as migraine headache, rather than being a specific clinical marker of fibromyalgia as might be suggested by previous SREP studies in the fibromyalgia population^34,50,51,61^. The current results showed a pattern of increasing subjective pain ratings during the SREP protocol in EM patients, but not in healthy controls. This pattern indicated a clear presence of SREP sensitization in EM patients relative to healthy individuals. According to published criteria for interpreting changes in VAS pain ratings, the increase of 1 point in VAS across the series of painful SREP stimuli observed exclusively in EM patients would be considered a significant change in pain^68^. This pain sensitization in response to the SREP protocol in EM patients is consistent with the lack of normal habituation to nociceptive stimulation previously observed in migraine patients^20,52^, even during the interictal period as in the current study^69^. Moreover, our current findings in EM patients are similar to the elevated SREP sensitization observed in fibromyalgia patients in our previous studies comparing fibromyalgia with both healthy individuals and rheumatoid arthritis patients as control conditions without CS^50,51^.

It should be noted that an elevated SREP sensitization response (positive slope across trials) was observed in the EM group despite there being no significant group differences (a) for each individual stimulus, (b) in the overall mean subjective perceived pain intensity across all SREP stimuli, (c) in the stimulus pressure used in the SREP protocol, and (d) in the initial VAS pain rating in the SREP series (between 3.2 and 3.5 in both groups). Thus, the SREP effect appeared to be a phenomenon distinct from overall evoked pain sensitivity. Indeed, the overlap in distribution for SREP responses between the two groups was minimal despite similar initial ratings, with only 2 EM patients showing lower SREP sensitization than the mean SREP index of the healthy group, and just 3 healthy individuals displaying greater SREP sensitization than the mean of the EM group. Higher levels of regular analgesic use and catastrophizing were observed in EM than in the healthy group, which is characteristic of patients with migraine^70,71^, but statistical control of these potential confounds did not alter the pattern of findings. In addition, levels of current pain intensity (PPI-MPQ), although not significantly different between groups, was significantly associated with SREP only in EM patients, highlighting its potential clinical utility. These levels of clinical pain were generally very low, since the presence of other comorbid pain conditions was an exclusion criterion and all testing was performed in the interictal phase. Statistical control for levels of clinical pain also did not alter the group differences observed in the SREP sensitization response.

Regarding the other evoked pain measures evaluated, there were no group differences in static pain measures (pain threshold and tolerance). This explains the absence of group differences in the mean stimulus pressure used for the SREP protocol, since this stimulus pressure was a function of both observed pain threshold and tolerance. In addition, the lack of significant differences between groups in the "stimulus-by-stimulus" comparisons of each VAS pain rating of the SREP series highlights the greater sensitivity of the dynamic SREP index as a clinically-relevant evoked pain measure relative to static evoked pain measures. There were also no group differences in the dynamic TSP measure, with a slight increase in pain perception during the TSP protocol of around 0.5 rating points observed in both EM and healthy individuals. The previous literature is mixed regarding findings expected for both static pain measures and TSP in migraine patients. In an exhaustive review, Nahman-Averbuch et al.^38^ noted studies in which pain threshold and tolerance were lower^72^, and TSP higher^9^ in migraine patients than in healthy individuals. However, other studies showed no difference in these pain measures^73–75^. Multiple factors can be considered as possible explanations for this lack of consistency: the modality of evoked pain stimulation (e.g., electrical, heat, cold, pressure stimulation), target body areas (e.g., face, neck, arm, or fingers), different type of migraine (e.g., episodic, chronic, with/without aura), and differences in sociodemographic variables (e.g., age or gender). Our specific sample was comprised of young women experiencing EM without aura versus young women without migraine, who did not present with other comorbid pain conditions, and whose recruitment was carried out in an academic environment. Therefore, it is possible that lower clinical symptom severity in our EM participants relative to other samples may partially explain the lack of group differences both in pain sensitivity (static pain measures) and TSP responses. Finally, we note that these traditional evoked pain measures have not proven to be especially sensitive to underlying CS processes^21,34,51^.

Logistic regression analyses showed a good accuracy of SREP in discriminating between EM patients and healthy individuals (75% with an optimal cut-off), although with slightly lower discriminative accuracy than in our previous studies with fibromyalgia patients (> 85%)^50,51^. In the current work, both sensitivity and specificity were relatively high (0.70 and 0.73, respectively). In comparison, accuracy of the more standard evoked pain measures in the present study for differentiating between EM patients and healthy individuals was relatively low, with both the combination of static measures (pain threshold and tolerance) and TSP showing an inability to distinguish between groups (accuracy ≈ 50%). These results are in line with the evidence above regarding inconsistent differences observed between migraine patients and healthy controls using these traditional pain measures in prior work^38^. Reasons for the clear SREP sensitization differences in the current study without corresponding differences in TSP in part could be explained by their methodological differences. While TSP protocols (including the one used in this study) do not usually adapt intensity of pain stimulation to individual´s pain sensitivity, pain intensity of stimuli used in the SREP protocol was adapted to each participant’s baseline pain responsiveness. In addition, TSP requires repeated pain stimulation at a stimulus frequency at 0.33 Hz or higher to generate wind up^48,49^, whereas SREP sensitization is elicited using a much lower frequency (0.03 Hz). Due to the differing stimulus parameters of SREP relative to TSP, it is hypothesized that they may reflect somewhat different underlying CS-related mechanisms. Though the exact nature of these mechanisms remains to be determined^51^, one possibility is that mechanisms underlying SREP could in part involve a reduction of descending inhibitory pain pathways (top-down CS). This is consistent with the differences in blood pressure-related hypoalgesia found between SREP and TSP (presence vs. absence of this pain inhibitory effect, respectively) in chronic pain patients^34,76^, which might help explain observed differences between SREP and TSP. While one might speculate that SREP taps specifically into top-down CS mechanisms that may be more relevant in migraine patients than does TSP (relatively more bottom-up mechanisms)^77^. However, the hypothesized impaired descending pain inhibition in EM patients would be expected also to increase pain sensitivity relative to controls, but no group differences in pain threshold and tolerance were observed. Because the SREP sensitization observed in EM patients (increase in VAS ≈ 1) was somewhat lower compared to the magnitude of SREP observed in fibromyalgia patients in our previous studies (≈ 1.5), we might speculate that there is greater dysfunction in the pain mechanisms underlying SREP sensitization in fibromyalgia patients relative to EM patients, as is suggested in some prior work regarding CS processes^78^. However, future research directly comparing fibromyalgia and EM patients is needed to support this suggestion.

The ROC analysis corroborated the high discriminatory power of SREP in differentiating between EM and healthy individuals. This analysis also revealed the three best SREP clinical cut-offs, based on Youden index values, for use in accurately identifying EM patients (SREP cut-off ≥ 0.50, 0.65, or 0.90). Although the three identified cut-offs showed the same overall diagnostic accuracy (75%), the specific cut-off selected should be determined based on the purpose (e.g., maximizing sensitivity in clinical settings versus maximizing specificity in research sample selection). For example, for detecting any signs of abnormal pain processing in a clinical setting, the lowest SREP cut-off (0.50) would be the best option as it displays the highest sensitivity (0.88, but with lower specificity of 0.62). In contrast, to optimize research samples and ensure clear dysfunction in pain processing by reducing false positives, the 0.90 SREP cut-off may be optimal (specificity of 0.90 but sensitivity of only 0.60). Applying a 0.50 SREP cut-off in the current sample, only 5 migraine patients are misclassified as healthy, but 15 healthy individuals are misclassified as migraineurs. In contrast, using a 0.90 SREP cut-off, 16 migraine patients are misclassified as healthy, but only 4 healthy individuals are misclassified.

Overall, the present findings provide evidence of the potential utility of SREP as a dynamic evoked pain index sensitive to CNS nociceptive alterations in migraine. The fact that elevated SREP was detected in migraine patients in this study even in the absence of current clinical pain (i.e., headache) highlights the potential of SREP for use in mechanistic studies. However, additional studies comparing SREP in other CS-related pain conditions (e.g., temporomandibular disorder) to patients with pain conditions believed not to have a significant CS component (i.e., not only healthy controls) are needed to justify conceptualizing SREP sensitization as a generic marker of CS-related processes.

It may be appropriate to consider including SREP in evoked pain QST protocols for research, and possibly clinical, purposes. QST usually includes several static and dynamic evoked pain measures with the purpose of characterizing pain modulation status in different pain conditions^24,32^. Currently, standard QST protocols include TSP as the sole CS index. However, our prior work comparing TSP versus SREP^51^ indicated that SREP sensitization is not redundant with TSP, although both appear to reflect CS-related changes. As suggested above, while TSP is sensitive to ascending facilitatory pain mechanisms, SREP might also be sensitive to descending inhibitory pain pathways. Thus, adding SREP as a dynamic evoked pain index to QST protocols could enhance the assessment of pain modulation related to CS processes. Thus, SREP sensitization might be useful in the discrimination of bottom-up versus top-down CS processes, and this could be useful clinically in the context of individualized treatment. Thus, if SREP primarily reflects top-down CS mechanisms, patients with greater SREP sensitization may obtain greater benefit from therapies that may enhance descending inhibition, such as physical exercise, psychological interventions, and medications enhancing neurotransmitters involved in pain inhibition (e.g., selective norepinephrine reuptake inhibitors (SNRIs)^6,79,80^.

The main limitations of this study are the following. The inclusion criteria for EM patients in this study might be considered too wide for our research objective. These criteria may have resulted in substantial heterogeneity in the EM sample, for example by including participants suffering from migraine attacks only 4 days per month and others suffering migraines 14 days per month. The young and relatively narrow age range of participants (21.10 ± 3.24 years old) could be another limitation, assuming that development of CS may evolve as the number and frequency of migraine attacks experienced increases^73^. However, based on the clinical interviews performed, the majority of patients reported first experiencing migraine attacks around menarche, and thus most already had a history of several years suffering from monthly migraine attacks. Another potential limitation was that there was no attempt to control for possible effects of menstrual phase, which might have influenced pain sensitivity^81^. Despite this, no participants were menstruating at the time of testing. The fact that the study only included women is another limitation of the study which precludes generalizing the results to men. The reasons for including only women were both the higher proportion of women affected by migraine^44^, and to avoid confounding effects due to sex differences in pain sensitivity^82^. Absence of blinding of the investigator ought to be noted as a limitation as well. Another potential limitation that might partially explain the differences found between SREP and TSP responses are the different assessment protocols^83^. Pain ratings were measured using a VAS for the SREP protocol, while in the TSP protocol, they were assessed verbally through NRS ratings, with VAS being impossible in the TSP protocol due to the high frequency of the pain stimuli. Furthermore, while SREP always caused a low-to-moderate pain due to the individual stimulus intensity calibration performed, the most common TSP protocols (including the current study) do not take into account individual differences in pain sensitivity, and thus the extent to which the TSP stimuli elicited pain across individuals may have differed substantially. Finally, the lack of information on the specific mechanisms underlying SREP sensitization limits the interpretation of results. Future studies comparing SREP with a more complete and standardized protocol such as the method of the German Research Network on Neuropathic Pain (DFNS)^25^ may be desirable to provide further information on the diagnostic utility of SREP sensitization. It might also be desirable to explore the potential implementation of the SREP protocol into the assessment of trigeminal sensitization specifically in migraine patients.

In summary, this study examined the potential utility of a dynamic evoked pain protocol, SREP, for detecting CNS-related nociceptive alterations in migraine. Previously, SREP had demonstrated its sensitivity to pain sensitization only in fibromyalgia patients. In the present study SREP displayed a significant overall accuracy of 71.3% in discriminating EM patients from healthy individuals, even though neither group was experiencing clinical pain at the time of testing. However, it should be noted that the SREP protocol is not proposed as a diagnostic tool, but rather as a marker sensitive to CS processes. Although EM patients displayed a broadly similar pain sensitization to that noted in fibromyalgia patients in previous work, its magnitude was somewhat lower in EM. Results showed that an SREP cut-off of 0.5 is sufficient to detect the presence of potential CS in young women suffering from migraine without aura with a diagnostic sensitivity of 0.88. Mechanisms underlying SREP remain unknown, and the possibility that SREP might tap into altered function in descending pain inhibitory pathways remains to be determined in future studies comparing responses between SREP and CPM protocols. Additional SREP studies targeting different CS-related pain conditions and control peripheral pain conditions are necessary to further evaluate the potential research and clinical utility of SREP, and to support its inclusion more broadly in QST protocols.

## References

[1] Stovner LJ (2007). "The global burden of headache: A documentation of headache prevalence and disability worldwide. Cephalalgia.

[2] de Tommaso M, Sciruicchio V (2016). Migraine and central sensitization: Clinical features, main comorbidities and therapeutic perspectives. Curr. Rheumatol. Rev..

[3] Dodick DW (2018). A phase-by-phase review of migraine pathophysiology. Headache J. Head Face Pain.

[4] Yunus MB (2015). Editorial review (thematic issue: An update on central sensitivity syndromes and the issues of nosology and psychobiology). Curr. Rheumatol. Rev..

[5] Kindler LL, Bennett RM, Jones KD (2011). Central sensitivity syndromes: Mounting pathophysiologic evidence to link fibromyalgia with other common chronic pain disorders. Pain Manag. Nurs..

[6] Harte SE, Harris RE, Clauw DJ (2018). The neurobiology of central sensitization. J. Appl. Biobehav. Res..

[7] Burstein R, Yarnitsky D, Goor-Aryeh I, Ransil BJ, Bajwa ZH (2000). An association between migraine and cutaneous allodynia. Ann. Neurol..

[8] de Tommaso M (2002). Abnormal brain processing of cutaneous pain in migraine patients during the attack. Neurosci. Lett..

[9] Weissman-Fogel I, Sprecher E, Granovsky Y, Yarnitsky D (2003). Repeated noxious stimulation of the skin enhances cutaneous pain perception of migraine patients in-between attacks: Clinical evidence for continuous sub-threshold increase in membrane excitability of central trigeminovascular neurons. Pain.

[10] Gracely RH, Schweinhardt P (2015). Programmed symptoms: Disparate effects united by purpose. Curr. Rheumatol. Rev..

[11] Staud R (2015). Cytokine and immune system abnormalities in fibromyalgia and other central sensitivity syndromes. Curr. Rheumatol. Rev..

[12] Biella G, Riva L, Sotgiu ML (1997). Interaction between neurons in different laminae of the dorsal horn of the spinal cord. A correlation study in normal and neuropathic rats. Eur. J. Neurosci..

[13] Quinn KP, Dong L, Golder FJ, Winkelstein BA (2010). Neuronal hyperexcitability in the dorsal horn after painful facet joint injury. Pain.

[14] Schwedt TJ, Dodick DW (2009). Advanced neuroimaging of migraine. Lancet Neurol..

[15] Montoya P, Pauli P, Batra A, Wiedemann G (2005). Altered processing of pain-related information in patients with fibromyalgia. Eur. J. Pain.

[16] Montoro CI, Duschek S, de Guevara CML, Reyes del Paso GA (2016). Patterns of cerebral blood flow modulation during painful stimulation in fibromyalgia: A transcranial doppler sonography study. Pain Med..

[17] Arendt-Nielsen L (2018). Assessment and manifestation of central sensitisation across different chronic pain conditions. Eur. J. Pain.

[18] Tracey I (2007). Neuroimaging of pain mechanisms. Curr. Opin. Support. Palliat. Care.

[19] Walitt B, Ceko M, Gracely J, Gracely R (2016). Neuroimaging of central sensitivity syndromes: Key insights from the scientific literature. Curr. Rheumatol. Rev..

[20] de Tommaso M, Sardaro M, Prudenzano MP, Lamberti P, Livrea P (2005). Lack of habituation of nociceptive evoked responses and pain sensitivity during migraine attack. Clin. Neurophysiol..

[21] Gracely RH, Grant MA, Giesecke T (2003). Evoked pain measures in fibromyalgia. Best Pract. Res. Clin. Rheumatol..

[22] Jakubowski M, Silberstein S, Ashkenazi A, Burstein R (2005). Can allodynic migraine patients be identified interictally using a questionnaire?. Neurology.

[23] Neblett R (2013). The Central Sensitization Inventory (CSI): Establishing clinically significant values for identifying central sensitivity syndromes in an outpatient chronic pain sample. J. Pain.

[24] Haanpää M (2011). NeuPSIG guidelines on neuropathic pain assessment. Pain.

[25] Rolke R (2006). Quantitative sensory testing in the German Research Network on Neuropathic Pain (DFNS): Standardized protocol and reference values. Pain.

[26] Attal N (2013). M Value of quantitative sensory testing in neurological and pain disorders: NeuPSIG consensus. Pain.

[27] Hansson P, Backonja M, Bouhassira D (2007). Usefulness and limitations of quantitative sensory testing: Clinical and research application in neuropathic pain states. Pain.

[28] Hashmi JA, Davis KD (2008). Effect of static and dynamic heat pain stimulus profiles on the temporal dynamics and interdependence of pain qualities, intensity, and affect. J. Neurophysiol..

[29] de la Coba P, Bruehl S, Garber J, Smith CA, Walker LS (2018). Is resolution of chronic pain associated with changes in blood pressure-related hypoalgesia?. Ann. Behav. Med..

[30] Eisenberg E, Midbari A, Haddad M, Pud D (2010). Predicting the analgesic effect to oxycodone by ‘static’and ‘dynamic’quantitative sensory testing in healthy subjects. Pain.

[31] Marcuzzi A, Wrigley PJ, Dean CM, Adams R, Hush JM (2017). The long-term reliability of static and dynamic quantitative sensory testing in healthy individuals. Pain.

[32] Arendt-Nielsen L, Yarnitsky D (2009). Experimental and clinical applications of quantitative sensory testing applied to skin, muscles and viscera. J. Pain.

[33] Boivie J (2003). Central pain and the role of quantitative sensory testing (QST) in research and diagnosis. Eur. J. Pain.

[34] de la Coba P, Bruehl S, Duschek S, Reyes del Paso GA (2018). Blood pressure-related pain modulation in fibromyalgia: Differentiating between static versus dynamic pain indicators. Int. J. Psychophysiol..

[35] Yarnitsky D (2010). Conditioned pain modulation (the diffuse noxious inhibitory control-like effect): Its relevance for acute and chronic pain states. Curr. Opin. Anesthesiol..

[36] Staud R, Vierck CJ, Cannon RL, Mauderli AP, Price DD (2001). Abnormal sensitization and temporal summation of second pain (wind-up) in patients with fibromyalgia syndrome. Pain.

[37] Kisler LB (2018). Do patients with interictal migraine modulate pain differently from healthy controls? A psychophysical and brain imaging study. Pain.

[38] Nahman-Averbuch H, Shefi T, Schneider VJ, Li D, Ding L, King CD, Coghill RC (2018). Quantitative sensory testing in patients with migraine: A systematic review and meta-analysis. Pain.

[39] Dodick D, Silberstein S (2006). Central sensitization theory of migraine: Clinical implications. Headache J. Head Face Pain.

[40] Centonze V (2004). Migraine, daily chronic headache and fibromyalgia in the same patient: an evolutive “continuum” of non organic chronic pain? About 100 clinical cases. Neurol. Sci..

[41] Sarchielli P, Di Filippo M, Nardi K, Calabresi P (2007). Sensitization, glutamate, and the link between migraine and fibromyalgia. Curr. Pain Headache Rep..

[42] Potvin S, Marchand S (2016). Pain facilitation and pain inhibition during conditioned pain modulation in fibromyalgia and in healthy controls. Pain.

[43] Staud R (2003). Temporal summation of pain from mechanical stimulation of muscle tissue in normal controls and subjects with fibromyalgia syndrome. Pain.

[44] Staud R, Robinson ME, Vierck CJ, Price DD (2003). Diffuse noxious inhibitory controls (DNIC) attenuate temporal summation of second pain in normal males but not in normal females or fibromyalgia patients. Pain.

[45] Potvin S, Paul-Savoie E, Morin M, Bourgault P, Marchand S (2012). Temporal summation of pain is not amplified in a large proportion of fibromyalgia patients. Pain Res. Treat..

[46] Stewart WF, Shechter A, Rasmussen BK (1994). Migraine prevalence. A review of population-based studies. Neurology.

[47] Wolfe F, Ross K, Anderson J, Russell IJ, Hebert L (1995). The prevalence and characteristics of fibromyalgia in the general population. Arthritis Rheum..

[48] Price DD, Hu JW, Dubner R, Gracely RH (1977). Peripheral suppression of first pain and central summation of second pain evoked by noxious heat pulses. Pain.

[49] Herrero JF, Laird JM, Lopez-Garcia JA (2000). Wind-up of spinal cord neurones and pain sensation: Much ado about something?. Prog. Neurobiol..

[50] de la Coba P, Bruehl S, Moreno-Padilla M, Reyes del Paso GA (2017). Responses to slowly repeated evoked pain stimuli in fibromyalgia patients: Evidence of enhanced pain sensitization. Pain Med..

[51] de la Coba P, Bruehl S, Galvez-Sánchez CM, Reyes del Paso GA (2018). Slowly repeated evoked pain as a marker of central sensitization in fibromyalgia: Diagnostic accuracy and reliability in comparison with temporal summation of pain. Psychosom. Med..

[52] Valeriani M (2003). Reduced habituation to experimental pain in migraine patients: A CO_2_ laser evoked potential study. Pain.

[53] Headache Classification Committee of the International Headache Society (IHS) The International Classification of Headache Disorders, 3rd edition. *Cephalalgia,* **38**, 1–211 (2018). 10.1177/0333102417738202 29368949

[54] Melzack R (1975). The McGill pain questionnaire. Pain.

[55] Masedo AI, Esteve R (2000). Some empirical evidence regarding the validity of the Spanish version of the McGill Pain Questionnaire (MPQ-SV). Pain.

[56] Rosenstiel AK, Keefe FJ (1983). The use of coping strategies in chronic low back pain patients: relationship to patient characteristics and current adjustment. Pain.

[57] Rodríguez Franco L, Cano-García FJ, Blanco-Picabia A (2004). Assessment of chronic pain strategies. Actas Españolas de Psiquiatría.

[58] Zigmond AS, Snaith RP (1983). The hospital anxiety and depression scale. Acta Psychiatr. Scand..

[59] Caro I, Ibáñez E (1992). Escala hospitalaria de ansiedad y depression. Su utilidad práctica en Psicología de la salud. Boletín de Psicología (Valencia).

[60] Quintana JM (2003). Evaluation of the psychometric characteristics of the Spanish version of the Hospital Anxiety and Depression Scale. Acta Psychiatr. Scand..

[61] de la Coba P, Bruehl S, Reyes del Paso GA (2019). Addition of slowly repeated evoked pain responses to clinical symptoms enhances fibromyalgia diagnostic accuracy. Pain Med..

[62] Reyes del Paso GA, Montoro C, Muñoz-Ladrón de Guevara C, Duschek S, Jennings JR (2014). The effect of baroreceptor stimulation on pain perception depends on the elicitation of the reflex cardiovascular response: Evidence of the interplay between the two branches of the baroreceptor system. Biol. Psychol..

[63] Goodin BR (2014). Temporal summation of pain as a prospective predictor of clinical pain severity in adults aged 45 years and above with knee osteoarthritis: Ethnic differences. Psychosom. Med..

[64] World Medical Association. WMA Declaration of Helsinki-Ethical principles for medical research involving human subjects (2013). 10.1001/jama.2013.281053 24141714

[65] Faul F, Erdfelder E, Buchner A, Lang AG (2009). Statistical power analyses using G* Power 3.1: Tests for correlation and regression analyses. Behav. Res. Methods.

[66] Altman DG, Bland JM (1994). Diagnostic tests. 1: Sensitivity and specificity. BMJ Br. Med. J..

[67] Hosmer Jr, D. W., Lemeshow, S., & Sturdivant, R. X. Syntax of referencing. In *Applied Logistic Regression*, 398 (Wiley, Hoboken, 2013)

[68] Carlsson AM (1983). Assessment of chronic pain. I. Aspects of the reliability and validity of the visual analogue scale. Pain.

[69] Coppola G, Di Lorenzo C, Schoenen J, Pierelli F (2013). Habituation and sensitization in primary headaches. J. Headache Pain.

[70] Bond DS (2015). Clinical pain catastrophizing in women with migraine and obesity. Headache J. Head Face Pain.

[71] Pires C, Sole E, Miro J (2013). Catastrophizing and pain impact in migraineurs. J. Headache Pain.

[72] Fernández-de-las-Peñas C (2010). Generalized neck-shoulder hyperalgesia in chronic tension-type headache and unilateral migraine assessed by pressure pain sensitivity topographical maps of the trapezius muscle. Cephalalgia.

[73] Buchgreitz L, Lyngberg AC, Bendtsen L, Jensen R (2006). Frequency of headache is related to sensitization: A population study. Pain.

[74] Drummond PD (1997). Photophobia and autonomic responses to facial pain in migraine. Brain J. Neurol..

[75] Zohsel K, Hohmeister J, Oelkers-Ax R, Flor H, Hermann C (2006). Quantitative sensory testing in children with migraine: Preliminary evidence for enhanced sensitivity to painful stimuli especially in girls. Pain.

[76] Chung OY, Bruehl S (2008). The impact of blood pressure and baroreflex sensitivity on wind-up. Anesth. Analg..

[77] Moulton EA, Burstein R, Tully S, Hargreaves R, Becerra L, Borsook D (2008). Interictal dysfunction of a brainstem descending modulatory center in migraine patients. PLoS ONE.

[78] Giamberardino MA (2016). Impact of migraine on fibromyalgia symptoms. J. Headache Pain.

[79] Bruehl S (2020). Are endogenous opioid mechanisms involved in the effects of aerobic exercise training on chronic low back pain?: A randomized controlled trial. Pain.

[80] Salomons TV, Moayedi M, Erpelding N, Davis KD (2014). A brief cognitive-behavioral intervention for pain reduces secondary hyperalgesia. Pain.

[81] Riley JL, Robinson ME, Wise EA, Price D (1999). A meta-analytic review of pain perception across the menstrual cycle. Pain.

[82] Bartley EJ, Fillingim RB (2013). Sex differences in pain: A brief review of clinical and experimental findings. Br. J. Anaesth..

[83] Firdous S (2017). A comparison of Numeric Pain Rating Scale (NPRS) and the Visual Analog Scale (VAS) in patients with chronic cancer-associated pain. J. Clin. Oncol..

